# SARS-CoV-2 spike HexaPro formulated in aluminium hydroxide and administered in an accelerated vaccination schedule partially protects Syrian Hamsters against viral challenge despite low neutralizing antibody responses

**DOI:** 10.3389/fimmu.2023.941281

**Published:** 2023-01-23

**Authors:** Dennis Christensen, Charlotta Polacek, Daniel J. Sheward, Leo Hanke, Gerald McInerney, Ben Murrell, Katrine Top Hartmann, Henrik Elvang Jensen, Julie Zimmermann, Gregers Jungersen, Kristin Engelhart Illigen, Louise Krag Isling, Carlota Fernandez-Antunez, Santseharay Ramirez, Jens Bukh, Gabriel Kristian Pedersen

**Affiliations:** ^1^ Center for Vaccine Research, Statens Serum Institut, Copenhagen, Denmark; ^2^ Virus Research and Development Laboratory, Department of Microbial Diagnostic and Virology, Statens Serum Institut, Copenhagen, Denmark; ^3^ Department of Microbiology, Tumor and Cell Biology, Karolinska Institutet, Stockholm, Sweden; ^4^ Department of Veterinary and Animal Sciences, University of Copenhagen, Copenhagen, Denmark; ^5^ Copenhagen Hepatitis C Program (CO-HEP), Department of Infectious Diseases, Copenhagen University Hospital, Hvidovre, Denmark; ^6^ Department of Immunology and Microbiology, Faculty of Health and Medical Sciences, University of Copenhagen, Copenhagen, Denmark

**Keywords:** SARS-CoV-2, alum, subunit vaccine, neutralizing antibodies, accelerated schedule

## Abstract

SARS-CoV-2 continues to pose a threat to human health as new variants emerge and thus a diverse vaccine pipeline is needed. We evaluated SARS-CoV-2 HexaPro spike protein formulated in Alhydrogel^®^ (aluminium oxyhydroxide) in Syrian hamsters, using an accelerated two dose regimen (given 10 days apart) and a standard regimen (two doses given 21 days apart). Both regimens elicited spike- and RBD-specific IgG antibody responses of similar magnitude, but *in vitro* virus neutralization was low or undetectable. Despite this, the accelerated two dose regimen offered reduction in viral load and protected against lung pathology upon challenge with homologous SARS-CoV-2 virus (Wuhan-Hu-1). This highlights that vaccine-induced protection against SARS-CoV-2 disease can be obtained despite low neutralizing antibody levels and suggests that accelerated vaccine schedules may be used to confer rapid protection against SARS-CoV-2 disease.

## Introduction

Several vaccines have been licensed against Severe Acute respiratory Syndrome Coronavirus 2 (SARS-CoV-2). Yet, the pandemic continues and the emergence of variant viruses resistant to first generation vaccines spurs a need for continued vaccine development. Antibody responses are a correlate of protection for many vaccines (1) and are sufficient to protect against SARS-CoV-2, although the levels and quality of antibody responses required for protection remain unclear (2). It is also unclear to what extent vaccine-induced T cells contribute to protection (3, 4) and thus, an increased understanding of correlates of protection against SARS-CoV-2 is needed.

SARS-CoV-2 vaccines relying on novel technologies, including RNA (5–7) and vectors (8, 9), have proved highly effective in controlling severe Covid-19 requiring hospitalizations and have changed the course of the pandemic. Yet, they put demand on the infrastructure, requiring establishment of cold-chains, which make them less attractive for mass-vaccination in developing countries. The vectored vaccines have also been associated with rare, but serious thrombotic events (10). With the risk of emerging SARS-CoV-2 variants that can escape immunity generated by first-generation Covid-19 vaccines, development of cheap, effective vaccines that can be produced at large scale and distributed under existing vaccine cold-chain distribution conditions (11, 12) should be investigated. Subunit vaccines can be highly effective against SARS-CoV-2 (13, 14), when delivered in the presence of an adjuvant. Adjuvants may influence affinity, specificity, magnitude and functional profile of B and T cell responses (15–17). To speed up SARS-CoV-2 vaccine development, a desired adjuvant should be safe, effective and available in large quantities. In this study we tested SARS-CoV-2 prefusion-stabilized HexaPro spike trimer (18) formulated in Alhydrogel^®^ (Aluminium Oxyhydroxide; AH), which fulfils these criteria being inexpensive and part of several licensed vaccines (19). Testing the vaccine in Syrian hamsters using an accelerated schedule (two doses given 10 days apart), low or undetectable neutralizing antibody responses were induced, yet the animals were protected against disease upon challenge with a high dose of SARS-CoV-2 (1.8 x 10^5^ TCID_50_) virus. This study suggests that vaccine-mediated protection against SARS-CoV-2 may be obtained, despite low neutralizing antibody responses, which may be used to further guide vaccine development.

## Material and methods

### Ethics statement

The Hamster studies were conducted in accordance with the regulations set forth by the National Committee for the Protection of Animals used for Scientific Purposes and in accordance with European Community Directive 2010/63/EU and have been approved by the governmental Animal Experiments Inspectorate under license 2020-15-0201-00554.

### Antigens and adjuvant

SARS-CoV-2 stabilized spike HexaPro trimer (18) antigen (Wuhan-Hu-1), and the RBD domain (RVQ-VNF) were produced by transient expression in Freestyle™ 293-F cells, as reported previously (20). The trimer was tested for human ACE2 (ACE-HM101, Kactus Biosystems) binding by ELISA. Aluminium oxyhydroxide (AH) (2% Alhydrogel^®^) was from CRODA Denmark (Frederikssund, Denmark).

### Hamsters

Male Syrian Golden Hamsters, nine weeks old, were ordered from Janvier and housed in the BSLII/III animal facilities at Statens Serum Institut, Denmark. The hamsters were maintained in a SPF room with controlled environment (20–23 ℃; relative humidity 55 ± 10%; 12/12 h light/dark cycle). The hamsters were housed in macrolon cages type IV (1820 cm^2^) with high lid (in total app. 30 cm. high) and Aspen bedding material from Tapvei. As enrichment the hamsters were offered Aspen bricks (Tapvei), Sizzelnest (Datesand), twisted paper roaps (“Diamond Twist” Envigo Teclad), tunnels and red houses made of macrolone. Irradiated sunflower seeds/corn grains/peanuts or bits of carrots were offered once a week. Pelleted diet (Envigo Teclad 2916) and tap water was provided ad libitum.

### Immunizations

Hamsters were administered two subcutaneous (s.c.) immunizations in the scruff of the neck with 10 days apart, using 10 μg recombinant SARS-CoV-2 spike ectodomain (HexaPro trimer) in TRIS buffer (pH 7.4) at a final volume of 200 μl per immunization. HexaPro trimer was administered alone or in AH used at a dose of 500 μg aluminium content.

### ELISA for antibody responses

Maxisorb Plates (Nunc) were coated overnight with 0.05 μg/well SARS-CoV-2 HexaPro trimer or RBD from the Wuhan-Hu-1 strain (produced in-house) or recombinant full spike protein from the B1.1.529 strain (rndsystems). Plates were incubated at 4 °C. After blocking, serum was added in PBS with 2% BSA, starting with a 30-fold dilution for antigen-specific IgG or IgG subclasses. Polyclonal anti-hamster HRP-conjugated secondary antibody (AB_2536572, Invitrogen), was diluted in PBS with 1% BSA. After 1 h of incubation, antigen-specific antibodies were detected using TMB substrate as described by the manufacturer (Kem-En-Tec Diagnostics), and the reaction was stopped with H_2_SO_4_. Sum of absorbances were calculated as described previously (21). Serum antibody avidity was tested using a sodium thiocyanate (NaSCN) ELISA assay as previously described (22). The avidity index was calculated as the ratio of IgG binding to RBD in the absence or presence of NaSCN and expressed as percentage binding, using a 90-fold serum dilution. A competition ELISA was performed to measure antibodies directed against the CR3022 epitope (23). Plates were coated with RBD overnight and blocked with PBS+ 2% BSA. Serum was added in dilution series and, following incubation, HRP-coupled CR3022 antibody (NBP2-90979H, Novusbio) was added before detection with TMB substrate.

### Neutralization assay

Neutralization of SARS-CoV-2 pseudo-particles was evaluated using a pseudotyped lentivirus neutralization assay with SARS-CoV-2 spike (Wu-Hu-1) and HEK293T cells engineered to express human ACE2, as previously described (24). ID50 values were estimated by fitting a logistic curve in Prism 5 (GraphPad Software), bounded between 0% and 100%, and interpolating the dilution at which luciferase expression was reduced by 50% relative to wells in the absence of serum. As a positive control in the assay we used an anti-spike nanobody (Ty1), which hinders RBD-ACE2 binding (24).

Neutralization of SARS-CoV-2 viruses was performed using the SARS-CoV-2/human/Denmark/DK-AHH1/2020 isolate cultured in Vero E6 cells, as previously described (25, 26). This isolate is closely related to the Wu-Hu-1 sequence (with E309K and D614G substitutions in the spike protein) In brief, SARS-CoV-2 virus at a multiplicity of infection (MOI) of 0.06 was incubated 1h at room temperature with serially diluted heat inactivated (at 56°C for 30 min) plasma (1/100 to 1/51200 dilution) at a 1:1 ratio. Following incubation, plasma/virus mixtures were added to naïve Vero E6 cells in quadruplicates in 96-well plates. After being incubated for 48h at 37°C and 5% CO_2_, cells were fixed and stained and single spots representing virus infected cells were counted by an Immunospot series 5 UV analyser, as described (25), and percent neutralization calculated. Neutralization curves were constructed and the ID50 of plasma was calculated using non-linear regression (Log [inhibitor] *vs* normalized response [variable slope]), using GraphPad Prism. A mouse derived SARS-CoV-2 spike neutralizing antibody (Sino Biological #40592-MM57, RRID: AB_2857935) was the positive control. Four replicates of a 1/800 dilution (1.25µg/mL) of the positive control were used for each plate, which gave an average of 81% neutralization. The investigators performing the neutralization assays were blinded to the experimental groups.

### Meso scale discovery ACE2 competition assay

The V-PLEX SARS-CoV-2 Panel 26 (ACE2) Kit was run according to the manufacturer´s instructions. Briefly, serum samples (diluted x25) were added to pre-coated V-plex plates followed by ACE2 binding detection using a Sector Imager 2400 system (Meso Scale Discovery).

### Antibody dependent complement deposition

ADCD was measured essentially as described previously (27). Biotinylated recombinant spike protein (2019-nCoV S1+S2 ECD-His, Sino Biological) was incubated with red 1.0 μm fluorescent neutravidin beads (Thermo Fisher) at 37°C with 1 μg of antigen per 1 μl of beads. After washing in 5% BSA-PBS, the coated beads were resuspended in 0.1% BSA + PBS and 10μl of beads was mixed with 10ul of serum samples (diluted x10) for 2 h at 37 °C to allow immune-complex formation. Subsequently, the beads were washed in 200 μl of PBS. Guinea pig complement (Cedarlane) was diluted 1:50 in dilution buffer (RPMI-1640 supplemented with 5 × 10-5 M 2-mercaptoethanol, 1% pyruvate, 1% HEPES, 1% (v/v) premixed penicillin-streptomycin solution (Invitrogen Life Technologies), 1 mM glutamine, and 10% (v/v) fetal calf serum (FCS) and incubated with the coated beads at 37°C for 15 minutes. The beads were then washed twice with 15 mM EDTA in PBS. A fluorescein-conjugated goat anti-guinea pig complement C3 (diluted 1:40 in PBS) was used to detect complement deposition. The samples were run in duplicate and analyzed on a BD Fortessa flow cytometer. Events were gated on single red fluorescent beads and displayed as percentage of C3 positive beads. Serum from naïve mice was used as a negative control (yielding 3.2% C3 positive beads) and serum from spike S-2P (Wu-Hu-1) immunized mice was used as a positive control (yielding 75% C3 positive beads) (28).

### SARS-CoV-2 challenge

SARS-CoV-2 virus was isolated from a Danish patient throat swab in PBS. The filtered (0.45 µm) material was propagated in a 24-well plate of Vero E6 cells (50 000 cells/well). The isolate was then passaged twice in Vero E6 (kind gift from Dr. Björn Meyer, Institut Pasteur, Paris, France) without cell culture acquired mutations (paired-end sequencing on miSeq platform, Illumina, CA, USA). TCID_50_-titers were determined by 7-fold serial dilutions on Vero E6 in a 96-well plate format. Titers were determined from wells showing cytopathic effect using the Reed-Muench method (29). All virus propagation was performed in Dulbecco’s modified Eagle’s media supplemented with 100U/ml Penicillin-Streptomycin (both from Gibco). The isolate SSI-H5 (GenBank accession number: ON809567) is closely related to the Wu-Hu-1 sequence, but with V367F and E990A substitutions in the spike protein and a G251V substitution in ORF3a). Hamsters were anesthetized with isofluorane and inoculated intranasally with 1.8 x 10^5^ TCID_50_ in 50ul of SARS-CoV-2. Weight and signs of disease was monitored until the study was terminated at 12 days post infection. Virus titres were determined in nasal washes (sampled by flushing with 350 µl phosphate buffered saline) at 5 days post challenge and in lungs at the time of termination.

### RT-qPCR for SARS-CoV-2 detection

Lungs were dissociated *via* GentleMACS (M-tubes, Miltenyi). Total RNA extractions were performed by MagNa Pure 96 system [Roche Molecular Biochemicals, Indianapolis, Indiana, United States (US)], using MagNA Pure LC DNA Isolation kit I lysis buffer. All oligonucleotides were synthesized by Eurofins Genomics. RT-qPCR was performed using 5 µl of resuspended RNA in a 25 µl reaction volume using the Luna^®^ Universal Probe One-Step RT-qPCR with Luna WarmStart^®^ RT Enzyme Mix (New England Biolabs) using 400 nM concentrations primers and 200 nM of probe. Primers and probes for the CoV-E gene target were as previously described (30). Cycling conditions were 55°C for 10 min, denaturation at 95°C for 3 min, and 45 cycles of 95°C (15 sec) and 58°C (30 sec). Reactions were carried out using a Lightcycler-480 Real-Time PCR System (Roche). To generate standard curves, synthetic SARS-CoV-2 RNA ctr1 (MT007544.1) (Twist Bioscience) of a known copy number was serially diluted. Results were expressed as log10-transformed numbers of genome equivalent copies per ml or g of sample. Detection of the subgenomic CoV-E target was performed as previously described (31) using 50°C for 10 min, 95°C for 3 min, and 45 cycles of 95°C (10 sec), 56°C (15 sec) and 72°C (5 sec). No standard curve was included for the subgenomic RNA detection and results are expressed as Ct value.

### Pathology

From animals euthanized on day 12 post challenge, the right diaphragmatic lung lobe was placed in histo-cassettes and fixed in 10% neutral buffered formalin for 24 hours before being transferred to ethanol. Paraffin embedded tissues were sectioned (4-μm) and stained with haematoxylin and eosin for histopathological examination. From each animal, two sections were present on the same slide, the whole of which was evaluated for infiltration of inflammatory cells (neutrophils and macrophages) and hyperplasia of type-II pneumocytes. In all sections the inflammatory reaction was scored as 0: absent, +: few sporadic inflammatory cells present, and ++: numerous accumulated inflammatory cells. The inflammatory cells were identified from their characteristic morphology (**Supplementary Figure 1A**). The presence of type-II pneumocyte hyperplasia was determined from the presence of this cell type laying the alveolar lumen in a row morphology (**Supplementary Figure 1B**). Immunohistochemically, type-II pneumocyte hyperplasia was documented by cytokeratin staining (32) (Mouse anti-cytokeratin clone AE1/AE3, DAKO M3515, diluted 1:8000). The investigators performing the histological examination were blinded to the experimental groups.

### Statistical analysis

Differences between groups were analysed by one-way ANOVA using Dunnet´s multiple comparisons test and the unvaccinated group as reference (GraphPad v8.2.1).

## Results

### Aluminium hydroxide adjuvanted SARS-CoV-2 spike trimer induces spike-specific antibodies but low levels of neutralizing responses in Syrian golden hamsters

**Figure 1:**
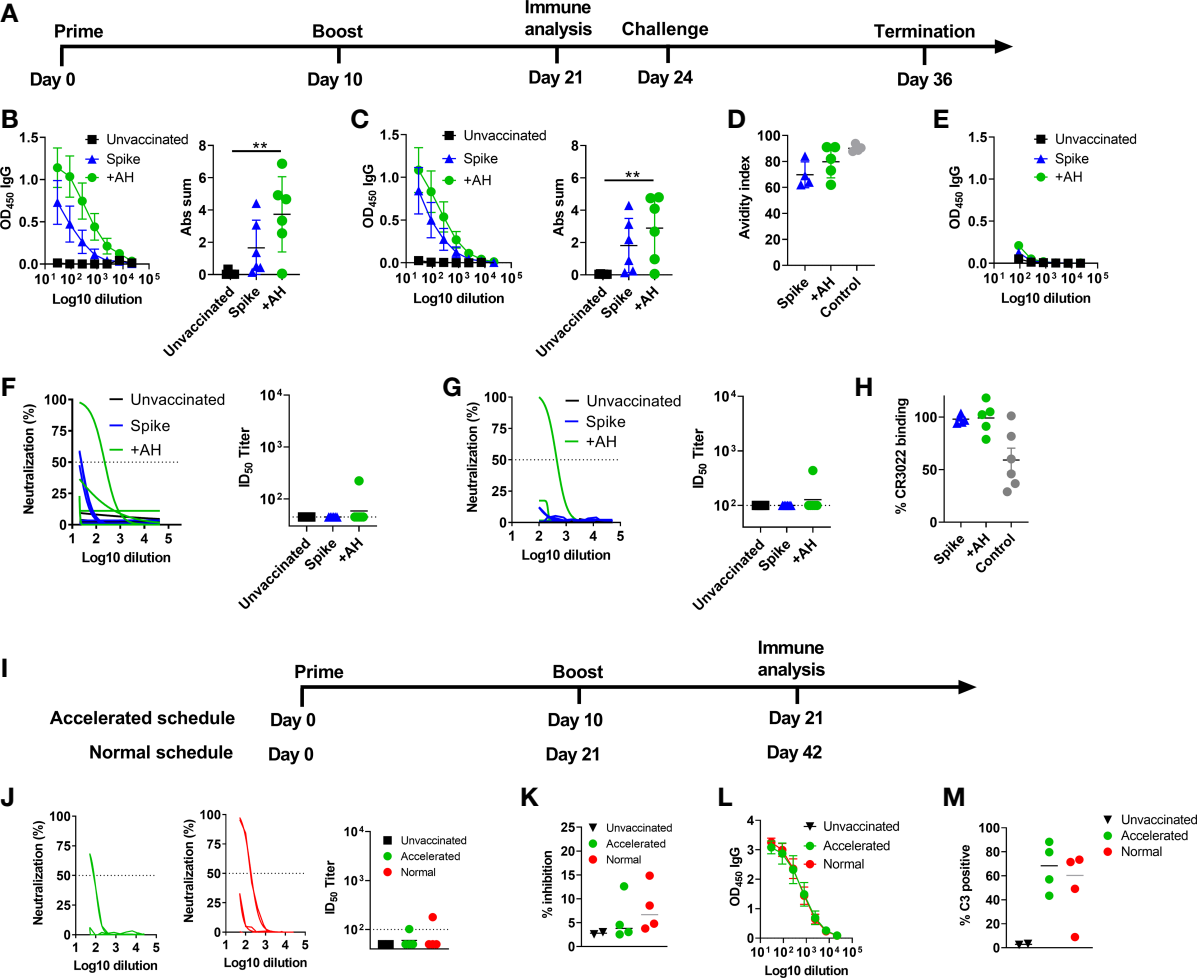
Aluminium hydroxide adjuvanted SARS-CoV-2 HexaPro spike protein elicits antibody responses with low neutralizing activity. Syrian Hamsters were immunized with two doses of spike trimer protein alone or formulated in aluminium hydroxide (AH). Serum was sampled at 11 days after the 2^nd^ immunization. **(A)** Timeline of the challenge experiment. Six hamsters were included in each group. **(B)** IgG antibody responses against spike protein from the homologous Wuhan-Hu-1 strain. **(C)** IgG antibody responses against the receptor binding domain (RBD) from the homologous Wuhan-Hu-1 strain. Mean ± SEM is displayed. Abs sum = sum of absorbances. **(D)** Antibody avidity against RBD. As a control, convalescent serum (day 14) from hamsters challenged with 1.8x10^5^ TCID_50_ of SARS-CoV-2 was included. **(E)** IgG antibody responses cross-reactive towards spike protein from the omicron (B.1.1.529) strain. **(F)** Neutralization of SARS-CoV-2 (Wuhan-Hu-1) by a pseudovirus neutralization assay and **(G)** a homotypic culture derived SARS-CoV-2 virus assay. **(H)** Serum **s**pecificity against the CR3022 epitope were tested in a competition ELISA. Data are displayed as reduction in CR3022 binding by serum from vaccinated groups compared to naïve serum. A positive control (mouse anti-spike serum) was included, which gives a 50% reduction in CR3022 binding. **(I)** Timeline of the experiment comparing accelerated and normal vaccine schedules. Syrian golden hamsters were immunized with two doses of spike trimer protein formulated in aluminium hydroxide given either as an accelerated regimen (10 days apart) and sampled at 11 days after the 2^nd^ immunization or as a normal regimen (21 days apart and sampled 21 days after the 2^nd^ immunization). Four hamsters were included in each vaccine group and two hamsters were non-immunized controls. **(J)** Neutralization of SARS-CoV-2 (Wuhan-Hu-1) by a homotypic culture derived SARS-CoV-2 virus assay. **(K)** ACE2 competition assay measuring serum antibodies towards the Wu-Hu-1 strain. **(L)** IgG antibody responses against spike protein from the homologous Wuhan-Hu-1 strain. **(M)** Antibody dependent complement deposition. Graphs displaying neutralization of culture derived SARS-CoV-2 virus **(G, J)** show the non-linear regression curve of SARS-CoV-2 virus inhibition normalized to non-treated controls (Y-axis) for the different plasma dilutions (X-axis). The dashed line highlights the 50% neutralization level. ID50 titers below the limit of detection (45 or 100) depending on sample availability are displayed as 45 or 100, respectively. An anti-spike nanobody (Ty1) was used as positive control in the pseudovirus assay, which gave 80% neutralization at 10ug/ml dilution, and a mouse derived SARS-CoV-2 spike neutralizing antibody was used as positive control in the homotypic culture derived virus assay, which gave an average of 81% neutralization at a 1/800 dilution (1.25µg/mL). Statistically significant differences are indicated by ** (one-way ANOVA using the unvaccinated group as reference, p<0.01). There was no statistically significant difference among groups if not otherwise indicated. Figures represent six hamsters per group.

The SARS-CoV-2 spike trimer (spike protein) mediates viral entry into host cells (33). The trimer protein is intrinsically metastable, but can be stabilized by proline substitutions. Homogenous spike trimers of the HexaPro variant (18) were produced and verified to bind to ACE2 (sFig1C). To evaluate the capacity of an AH-adjuvanted spike protein-based vaccine to protect against SARS-CoV-2, we used a Syrian golden hamster (*Mesocricetus auratus*) model, using an accelerated regimen, informed by murine studies where AH-adjuvanted spike protein induced neutralizing antibody responses after a single immunization (34). Hamsters were immunized twice (10 days apart) with 10µg of spike protein *via* the subcutaneous route and challenged 14 days after the second immunization (**Figure 1A**). Spike protein was administered either alone or given in AH. Prior to challenge (11 days post the second dose), serum IgG antibody responses were measured against the full-length spike protein and the RBD, the main determinant for neutralization. AH enhanced both total spike protein-specific antibody titres (**Figure 1B**) and to a lesser extent RBD-specific antibody titres (**Figure 1C**) compared to the spike trimer used without adjuvant. Antibody avidity, measured by sodium thiocyanate (NaSCN) ELISA, was not significantly different between the AH-adjuvanted and the spike alone group (**Figure 1D**). Although spike-specific IgG antibody responses could be detected against the homologous Wuhan-Hu-1 strain, we detected very low IgG responses reactive against spike from the omicron (B.1.1.529) variant (**Figure 1E**). To investigate if the vaccine-elicited antibodies were capable of neutralizing SARS-CoV-2, we performed a homotypic SARS-CoV-2 pseudovirus neutralization assay, but found low neutralizing capacity of the sera, with detectable responses in only one of the six hamsters in the AH group (ID50 of 1/223) (**Figure 1F**). Similar results were obtained in the assay using homotypic culture derived SARS-CoV-2 viruses for the neutralization assays, with the pseudovirus assay positive animal having an ID50 of 1/436. All other plasma samples tested at a 1/100 or higher dilutions lacked activity in this assay (**Figure 1G**). The RBD-specific monoclonal antibody CR3022 may drive antibody effector functions including antibody-dependent cellular phagocytosis (ADCP), but was reported not to neutralize SARS-CoV-2 (35–37), although a recent study suggested an unusual mechanism of neutralization (destruction of the prefusion trimer), which was only picked up under certain neutralizing assay conditions (23). We did not observe inhibition of CR3022 binding to RBD by serum from the AH-adjuvanted spike trimer group or the unadjuvanted group in a competition ELISA (**Figure 1H**), suggesting that the vaccine did not induce antibody responses capable of competing with CR3022 binding.

To investigate if the low neutralizing antibody responses were due to the accelerated vaccine schedule, we repeated the study, but included an additional group receiving a standard vaccine regimen of two vaccine doses given 21 days apart. For this group, we evaluated neutralizing antibody responses three weeks after the last immunization to allow for maturation of the antibody response (**Figure 1I**). Interestingly, also this group had low neutralizing antibody responses with only one of four hamsters displaying neutralization in the homotypic culture derived SARS-CoV-2 virus neutralization assay (**Figure 1J**). Similarly, low antibody responses were found in an ACE2-competition assay (**Figure 1K**), both in the accelerated and normal schedule groups. Furthermore, although all hamsters had detectable anti-spike IgG, the antibody levels were similar between the accelerated and standard vaccine schedule (**Figure 1L**). Thus, the AH-adjuvanted spike protein vaccine elicited low neutralizing antibody responses regardless of the tested vaccine schedules. We then investigated if the elicited spike-specific antibody responses were able to activate complement, as another potential antibody-mediated effector mechanism. Interestingly, sera from both the accelerated and normal vaccine schedule activated complement, as measured by C3 deposition (**Figure 1M** and **Supplementary Figure 2**).

### Aluminium hydroxide adjuvanted spike trimer vaccine protects against disease following SARS-CoV-2 challenge

**Figure 2:**
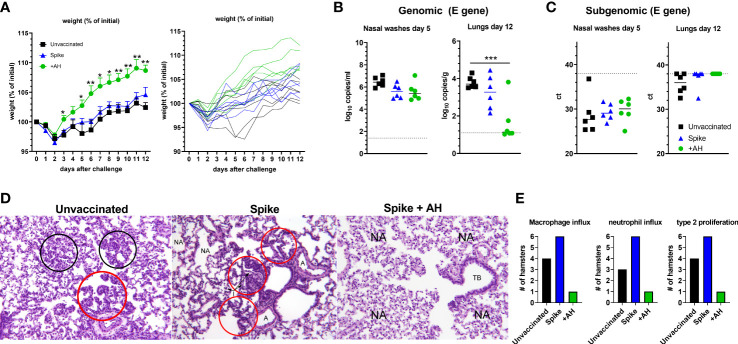
Spike protein adjuvanted with aluminium hydroxide (AH) protects against challenge with SARS-CoV-2. Syrian golden hamsters were immunized with two doses of spike trimer protein alone or formulated in AH and challenged intranasally with 1.8x10^5^ TCID_50_ of SARS-CoV-2. **(A)** Percent weight change. Individual hamsters are displayed in the right panel. **(B)** Viral load by qPCR in the nasal washes at 5 dpi (left panel) and in lungs at 12 dpi (right panel) measured by qPCR against the E-gene. **(C)** Subgenomic RNA in the nasal washes at 5 dpi (left panel) and in lungs at 14 dpi (right panel). Statistically significant differences are indicated by *** (one-way ANOVA using the unvaccinated group as reference, p<0.001). There was no statistically significant difference among groups if not otherwise indicated. **(D)** Right diaphragmatic lung lobe were fixed in 10% formalin, cut and stained with Hematoxylin and Eosin (HE) to examine pulmonary pathology at 12 dpi. Representative stainings (obj. x 10) of unvaccinated animals (left panel) show influx of inflammatory cells (black circles). Red circles show type II pneumocyte hyperplasia. Spike vaccinated animals (middle panel) had inflammatory cells present within alveoli (I) and type II pneumocyte hyperplasia (red circles). Few normal alveoli (NA) are present **(A)** normal arteria. Spike + AH vaccinated animals (right panel) had normal alveoli (NA) and normal terminal bronchiole (TB). **(E)** Number of hamsters in each group having influx of the indicated inflammatory cells and type II pneumocyte proliferation, respectively (n =3 (mock) to 6 (vaccinated and/or challenged) hamsters per group).

Hamsters that had received the accelerated vaccine schedule were challenged intranasally with a high dose of SARS-CoV-2 virus (1.8 x 10^5^ TCID_50_) of an early Danish isolate (SARS-CoV-2/Hu/DK/SSI-H5) to investigate if the vaccine was capable of protecting against disease despite the low neutralizing antibody responses. All infected groups had a transient weight loss at two days post infection. Subsequently, hamsters in the AH vaccinated group rapidly gained weight, whilst the infection-only group had reduced weight until six days post virus inoculation [98% of starting weight on average compared to 105% in the AH adjuvanted group, significantly different (p<0.05)] and recovered slower than the AH vaccinated animals (**Figure 2A**). At five days post challenge, qPCR of the SARS-CoV-2 E gene detected virus in nasal washes from all challenged animals, although there was a tendency towards lower virus titres in the group vaccinated with AH-adjuvanted spike protein and unadjuvanted spike protein than in unvaccinated animals (not significant, p = 0.05 and 0.06, respectively, **Figure 2B**). Animals that received AH-adjuvanted vaccine had significantly lower lung virus titres at 12 days post challenge compared to the unvaccinated group (p<0.001) and four of six animals had no detectable virus. There was also a tendency towards lower virus titres in the unadjuvanted vaccine group compared to unvaccinated controls (not significant, p = 0.47). An RT-qPCR to detect E-gene subgenomic RNA, as a surrogate of active viral infection, suggested that there was no actively replicating virus in the lungs of hamsters vaccinated with AH-adjuvanted spike, whilst subgenomic RNA could be detected in four out of six animals in the unvaccinated group (**Figure 2C**). The SARS-CoV-2 challenge further boosted antibody responses against spike protein from the homologous Wuhan-Hu-1 strain (**Supplementary Figure 3A**). Post-infection sera also reacted towards spike protein from the omicron (B.1.1.529 variant) (**Supplementary Figure 3B**).

SARS-CoV-2 causes lung lesions in hamsters comparable to those of human disease, including marked infiltration with neutrophils and mononuclear cells and hyperplasia of type-II pneumocytes (38, 39). To examine if the vaccine could protect against pulmonary pathology, we examined the challenged hamsters for signs of lung lesions at 12 days post infection. In the unvaccinated group and the spike-alone group, there were clear signs of remaining inflammatory infiltrates consisting of large numbers of neutrophils and macrophages (**Figures 2D, E** and **Supplementary Figure 1B**). We also observed prominent type-II pneumocyte hyperplasia along the alveolar lining (**Figures 2D, E**). In the AH-adjuvanted group, one hamster had signs of lung inflammation at 12 days post infection. Notably, this animal had the lowest spike-specific and RBD-specific IgG antibody responses (**Figure 1**) and non-detectable neutralization titres, suggesting low vaccine response in this animal. The remaining five hamsters in the AH-adjuvanted group had no signs of lung pathology.

## Discussion

Safe and effective vaccines that can be produced at large scale and are suitable for use in developing countries are needed to protect against emerging SARS-CoV-2 variants. Subunit vaccines based on proteins, are used successfully against other infections, e.g. hepatitis B and Varicella zoster virus, and are important components of the SARS-CoV-2 vaccine pipeline. Recently, the Matrix-M™ adjuvanted spike-protein based subunit vaccine (NVX-CoV2373) (40) was granted emergency use authorization and another spike-based vaccine, adjuvanted with alum + CpG1018 (SCB-2019) is currently in phase 3 clinical trials (41).

Syrian hamsters are considered a relevant model for human SARS-CoV-2 infection, demonstrating similar viral kinetics, disease course and immune responses as seen in patients with Covid-19 (39, 42) and, as opposed to in transgenic mice, in which infection of the central nervous system (CNS) is the major manifestation of the disease (43), disease in Syrian hamsters predominantly affects the lower respiratory tract (39). Previous vaccine studies in Syrian hamsters demonstrated high neutralizing antibody titres and protection against SARS-CoV-2 challenge by adenovirus serotype 26 and chimpanzee adenovirus vectors expressing stabilized SARS-CoV-2 spike protein (44, 45). Protein subunit vaccines composed of spike ectodomain protein with a nitrogen bisphosphonate–modified zinc-aluminum hybrid adjuvant (46) and spike (S-2P) protein adjuvanted with AH+CpG1018 also elicited high neutralizing antibody responses and protected against challenge in Syrian hamsters (47). In contrast, a DNA vaccine based on SARS-CoV-2 full-length spike failed to protect against lung pathology in a Syrian hamster model. This vaccine also failed to induce neutralizing antibody responses, despite eliciting anti-RBD antibody responses (48).

Here we have demonstrated that the pre-fusion stabilized (HexaPro) SARS-CoV-2 spike protein (18) formulated with AH and given in an accelerated vaccine schedule protects against lower respiratory tract infection and pathology upon SARS-CoV-2 challenge in Syrian hamsters. Notably, the vaccine elicited antibodies against spike RBD, but serum antibody responses had low neutralizing activity *in vitro*, with only one of six animals mounting neutralizing antibody responses. This animal also had anti-RBD antibody responses and was protected against SARS-CoV-2 challenge with no virus detected in the lungs. Using a similar AH-adjuvanted pre-fusion stabilized (S-2P) we found high neutralizing antibody responses 42 days after a single immunization in mice (34) and previous studies have demonstrated that the HexaPro antigen adjuvanted with AH elicits robust neutralizing antibody responses in mice when using a standard vaccine regimen (two doses given three weeks apart and evaluation of responses two weeks later) (49). In contrast, more recent studies have demonstrated that alum adjuvanted recombinant spike or RBD-based vaccines are poor at inducing neutralizing antibody responses, particularly in Syrian hamsters (50, 51). In the present study, AH-adjuvanted spike protein was poor at inducing neutralizing antibody responses in Syrian hamsters, regardless of whether the vaccine was administered in an accelerated schedule (10 days apart) or a standard schedule (21 days apart). This suggests that alum is not an optimal adjuvant for eliciting SARS-CoV-2 neutralizing antibody responses in hamsters. Nonetheless, this study suggests that protection against SARS-CoV-2 disease may be obtained with vaccines offering low neutralizing antibody activity. Similarly, it has previously been demonstrated that vaccine-induced protection against disease in humans may occur prior to observation of neutralizing antibodies. It is possible that vaccine-induced neutralizing antibodies, despite being below the limit of detection, are sufficient for protection against SARS-CoV-2 disease or that the vaccine facilitated protection by other mechanisms than neutralizing antibodies, e.g. non-neutralizing antibodies acting through Fc receptor functions (52). Notably, both the accelerated and normal vaccine schedule elicited serum antibodies capable of activating antibody dependent complement deposition (ADCD), which could potentially contribute to protection. It is also possible that the alum-adjuvanted vaccine elicited protective T cell responses (53) and reviewed in (54), which is a focus for further studies.

Vaccines against SARS-CoV-2 aim at inducing neutralizing antibodies, but if this is required for vaccine-induced protection against SARS-CoV-2 infection and disease is now questioned. We found that an adjuvanted spike protein subunit vaccine elicited very low neutralizing antibody responses, but still protected against SARS-CoV-2 disease. This suggests that vaccine-induced protection may be obtained with vaccines that are poor at inducing neutralizing antibody responses. Aluminium hydroxide adjuvanted spike subunit vaccines can easily be produced at low cost and in large quantities and are suitable for mass-vaccination. However, the present study suggests that aluminium hydroxide is not an optimal adjuvant for elicitation of SARS-CoV-2 neutralizing antibody responses when evaluated in the hamster model.

## Data availability statement

The raw data supporting the conclusions of this article will be made available by the authors, without undue reservation.

## Ethics statement

The animal study was reviewed and approved by the Danish Governmental Animal Experiments Inspectorate.

## Author contributions

DC, CP, GP contributed to conception and design of the study. DC and GP wrote the first draft of the manuscript, CP, DS, LH, KH, CF and SR performed experiments. All authors contributed to manuscript revision, read, and approved the submitted version

## Supplementary material

Supplementary Figure 1
**(A)** Representative stainings of lungs from unvaccinated hamsters at 12 days post infection with SARS-CoV-2. Influx of different inflammatory cells are pointed out. Neutrophils (arrows) and macrophages (arrowheads) are observed. Obj. x 40. **(B)** Representative stainings of unvaccinated hamsters at 12 days post infection with SARS-CoV-2 for presence of type II pneumocyte hyperplasia, demonstrated by immunostaining of cytokeratin. Obj. x 25. **(C)** Confirmation of antigen (HexaPro) binding to ACE2 by ELISA.

Supplementary Figure 2 Syrian golden hamsters were immunized with two doses of spike trimer protein alone or formulated in AH and challenged intranasally with 1.8x10^5^ TCID_50_ of SARS-CoV-2. IgG antibody responses were measured in serum from 12 days post-infection against full spike protein from **(A)** the homologous Wuhan-Hu-1 strain and **(B)** the omicron (B.1.1.529. variant.

Supplementary Figure 3 Syrian golden hamsters were immunized with two doses of spike trimer protein formulated in aluminium hydroxide given either as an accelerated regimen (10 days apart) and sampled at 11 days after the 2^nd^ immunization or as a normal regimen (21 days apart and sampled 21 days after the 2^nd^ immunization. Antibody dependent complement deposition was measured by coating beads with spike protein. Sera from vaccinated or non-vaccinated hamsters and complement was added and antibody-dependent complement deposition was assayed by measuring C3 deposition by flow cytometry.

## References

[1] Plotkin SA . Correlates of protection induced by vaccination. Clin Vaccine Immunol (2010) 17(7):1055–65. doi: 10.1128/CVI.00131-10 PMC2897268 20463105

[2] Corbett KS Nason MC Flach B Gagne M O’Connell S Johnston TS . Immune correlates of protection by mrna-1273 vaccine against sars-Cov-2 in nonhuman primates. Science (2021) 373(6561):. doi: 10.1126/science.abj0299 PMC8449013 34529476

[3] Rydyznski Moderbacher C Ramirez SI Dan JM Grifoni A Hastie KM Weiskopf D . Antigen-specific adaptive immunity to sars-Cov-2 in acute covid-19 and associations with age and disease severity. Cell (2020) 183(4):996–1012.e19. doi: 10.1016/j.cell.2020.09.038 33010815 PMC7494270

[4] Bange EM Han NA Wileyto P Kim JY Gouma S Robinson J . Cd8+ T cells contribute to survival in patients with covid-19 and hematologic cancer. Nat Med (2021) 27(7):1280–9. doi: 10.1038/s41591-021-01386-7 PMC8291091 34017137

[5] Jackson LA Anderson EJ Rouphael NG Roberts PC Makhene M Coler RN . An mrna vaccine against sars-Cov-2 - preliminary report. N Engl J Med (2020) 383(20):1920–31. doi: 10.1056/NEJMoa2022483 PMC7377258 32663912

[6] Erasmus JH Khandhar AP O'Connor MA Walls AC Hemann EA Murapa P . An alphavirus-derived replicon rna vaccine induces sars-Cov-2 neutralizing antibody and T cell responses in mice and nonhuman primates. Sci Transl Med (2020) 12(555). doi: 10.1126/scitranslmed.abc9396 PMC7402629 32690628

[7] Polack FP Thomas SJ Kitchin N Absalon J Gurtman A Lockhart S . Safety and efficacy of the Bnt162b2 mrna covid-19 vaccine. N Engl J Med (2020) 383(27):2603–15. doi: 10.1056/NEJMoa2034577 PMC7745181 33301246

[8] Zhu FC Guan XH Li YH Huang JY Jiang T Hou LH . Immunogenicity and safety of a recombinant adenovirus type-5-Vectored covid-19 vaccine in healthy adults aged 18 years or older: A randomised, double-blind, placebo-controlled, phase 2 trial. Lancet (2020) 396(10249):479–88. doi: 10.1016/S0140-6736(20)31605-6 PMC7836858 32702299

[9] Voysey M Clemens SAC Madhi SA Weckx LY Folegatti PM Aley PK . Safety and efficacy of the Chadox1 ncov-19 vaccine (Azd1222) against sars-Cov-2: An interim analysis of four randomised controlled trials in Brazil, south Africa, and the uk. Lancet (2021) 397(10269):99–111. doi: 10.1016/S0140-6736(20)32661-1 33306989 PMC7723445

[10] Schultz NH Sørvoll IH Michelsen AE Munthe LA Lund-Johansen F Ahlen MT . Thrombosis and thrombocytopenia after Chadox1 ncov-19 vaccination. N Eng J Med (2021) 384(22):2124–30. doi: 10.1056/NEJMoa2104882 PMC8112568 33835768

[11] Who guidelines on stability evaluation (Accessed 10 December 2021).

[12] Who generic preferred product profile for vaccines (Accessed 10 December 2021).

[13] Heath PT Galiza EP Baxter DN Boffito M Browne D Burns F . Safety and efficacy of nvx-Cov2373 covid-19 vaccine. N Eng J Med (2021) 385(13):1172–83. doi: 10.1056/NEJMoa2107659 PMC8262625 34192426

[14] Keech C Albert G Cho I Robertson A Reed P Neal S . Phase 1-2 trial of a sars-Cov-2 recombinant spike protein nanoparticle vaccine. N Engl J Med (2020) 383(24):2320–32. doi: 10.1056/NEJMoa2026920 PMC7494251 32877576

[15] Khurana S Verma N Yewdell JW Hilbert AK Castellino F Lattanzi M . Mf59 adjuvant enhances diversity and affinity of antibody-mediated immune response to pandemic influenza vaccines. Sci Transl Med (2011) 3(85):85ra48. doi: 10.1126/scitranslmed.3002336 PMC3501657 21632986

[16] Leroux-Roels G Marchant A Levy J Van Damme P Schwarz TF Horsmans Y . Impact of adjuvants on Cd4(+) T cell and b cell responses to a protein antigen vaccine: Results from a phase ii, randomized, multicenter trial. Clin Immunol (2016) 169:16–27. doi: 10.1016/j.clim.2016.05.007 27236001

[17] Del Giudice G Rappuoli R Didierlaurent AM . Correlates of adjuvanticity: A review on adjuvants in licensed vaccines. Semin Immunol (2018) 39:14–21. doi: 10.1016/j.smim.2018.05.001 29801750

[18] Hsieh C-L Goldsmith JA Schaub JM DiVenere AM Kuo H-C Javanmardi K . Structure-based design of prefusion-stabilized sars-Cov-2 spikes. Science (2020) 369:1501–5. 10.1126/science.abd0826 PMC7402631 32703906

[19] HogenEsch H O'Hagan DT Fox CB . Optimizing the utilization of aluminum adjuvants in vaccines: You might just get what you want. NPJ Vaccines (2018) 3:51. doi: 10.1038/s41541-018-0089-x 30323958 PMC6180056

[20] Sheward DJ Mandolesi M Urgard E Kim C Hanke L Perez Vidakovics L . Beta rbd boost broadens antibody-mediated protection against sars-Cov-2 variants in animal models. Cell Rep Med (2021) 2(11):100450. doi: 10.1016/j.xcrm.2021.100450 34723224 PMC8536561

[21] Hartman H Wang Y Schroeder HW Jr. Cui X . Absorbance summation: A novel approach for analyzing high-throughput Elisa data in the absence of a standard. PloS One (2018) 13(6):e0198528. doi: 10.1371/journal.pone.0198528 29883460 PMC5993274

[22] Pedersen GK Hoschler K Oie Solbak SM Bredholt G Pathirana RD Afsar A . Serum igg titres, but not avidity, correlates with neutralizing antibody response after H5n1 vaccination. Vaccine (2014) 32(35):4550–7. doi: 10.1016/j.vaccine.2014.06.009 24950357

[23] Huo J Zhao Y Ren J Zhou D Duyvesteyn HME Ginn HM . Neutralization of sars-Cov-2 by destruction of the prefusion spike. Cell Host Microbe (2020) 28(3):445–54.e6. doi: 10.1016/j.chom.2020.06.010 32585135 PMC7303615

[24] Hanke L Vidakovics Perez L Sheward DJ Das H Schulte T Moliner-Morro A . An alpaca nanobody neutralizes sars-Cov-2 by blocking receptor interaction. Nat Commun (2020) 11(1):4420. doi: 10.1038/s41467-020-18174-5 32887876 PMC7473855

[25] Ramirez S Fernandez-Antunez C Galli A Underwood A Pham LV Ryberg LA . Overcoming culture restriction for sars-Cov-2 in human cells facilitates the screening of compounds inhibiting viral replication. Antimicrob Agents Chemother (2021) 65(7):e0009721. doi: 10.1128/AAC.00097-21 33903110 PMC8406809

[26] Underwood AP Solund C Fernandez-Antunez C Villadsen SL Winckelmann AA Bollerup S . Neutralisation titres against sars-Cov-2 are sustained 6 months after onset of symptoms in individuals with mild covid-19. EBioMedicine (2021) 71:103519. doi: 10.1016/j.ebiom.2021.103519 34419923 PMC8375401

[27] Fischinger S Fallon JK Michell AR Broge T Suscovich TJ Streeck H . A high-throughput, bead-based, antigen-specific assay to assess the ability of antibodies to induce complement activation. J Immunol Methods (2019) 473:112630. doi: 10.1016/j.jim.2019.07.002 31301278 PMC6722412

[28] Christensen D Polacek C Sheward DJ Hanke L Moliner-Morro A McInerney G . Protection against sars-Cov-2 transmission by a parenteral prime-intranasal boost vaccine strategy. EBioMedicine (2022) 84:104248. doi: 10.1016/j.ebiom.2022.104248 36088218 PMC9448948

[29] Reed LJ Muench H . A simple method of estimating fifty per cent Endpoints12. Am J Epidemiol (1938) 27(3):493–7. doi: 10.1093/oxfordjournals.aje.a118408%J American Journal of Epidemiology

[30] Corman VM Landt O Kaiser M Molenkamp R Meijer A Chu DK . Detection of 2019 novel coronavirus (2019-ncov) by real-time rt-pcr. Euro Surveill (2020) 25(3):2000045. doi: 10.2807/1560-7917.ES.2020.25.3.2000045 31992387 PMC6988269

[31] Wolfel R Corman VM Guggemos W Seilmaier M Zange S Muller MA . Virological assessment of hospitalized patients with covid-2019. Nature (2020) 581(7809):465–9. doi: 10.1038/s41586-020-2196-x 32235945

[32] Grossi AB Leifsson PS Jensen HE Vainer B Iburg T . Histologic and immunohistochemical classification of 41 bovine adrenal gland neoplasms. Vet Pathol (2013) 50(3):534–42. doi: 10.1177/0300985812469638 23242804

[33] Jiang S Hillyer C Du L . Neutralizing antibodies against sars-Cov-2 and other human coronaviruses. Trends Immunol (2020) 41(5):355–9. doi: 10.1016/j.it.2020.03.007 PMC7129017 32249063

[34] Wørzner K Sheward DJ Schmidt ST Hanke L Zimmermann J McInerney G . Adjuvanted sars-Cov-2 spike protein elicits neutralizing antibodies and Cd4 T cell responses after a single immunization in mice. EBioMedicine (2021) 63:103197. doi: 10.1016/j.ebiom.2020.103197 33422991 PMC7808923

[35] Wrobel AG Benton DJ Hussain S Harvey R Martin SR Roustan C . Antibody-mediated disruption of the sars-Cov-2 spike glycoprotein. Nat Commun (2020) 11(1):5337. doi: 10.1038/s41467-020-19146-5 33087721 PMC7577971

[36] Wu NC Yuan M Bangaru S Huang D Zhu X Lee CD . A natural mutation between sars-Cov-2 and sars-cov determines neutralization by a cross-reactive antibody. PloS Pathog (2020) 16(12):e1009089. doi: 10.1371/journal.ppat.1009089 33275640 PMC7744049

[37] Lv Z Deng YQ Ye Q Cao L Sun CY Fan C . Structural basis for neutralization of sars-Cov-2 and sars-cov by a potent therapeutic antibody. Science (2020) 369(6510):1505–9. doi: 10.1126/science.abc5881 PMC7402622 32703908

[38] Rosenke K Meade-White K Letko M Clancy C Hansen F Liu Y . Defining the Syrian hamster as a highly susceptible preclinical model for sars-Cov-2 infection. Emerging Microbes Infections (2020) 9(1):2673–84. doi: 10.1080/22221751.2020.1858177 PMC7782266 33251966

[39] Imai M Iwatsuki-Horimoto K Hatta M Loeber S Halfmann PJ Nakajima N . Syrian Hamsters as a small animal model for sars-Cov-2 infection and countermeasure development. Proc Natl Acad Sci U.S.A. (2020) 117(28):16587–95. doi: 10.1073/pnas.2009799117 PMC7368255 32571934

[40] Heath PT Galiza EP Baxter DN Boffito M Browne D Burns F . Safety and efficacy of nvx-Cov2373 covid-19 vaccine. N Engl J Med (2021) 385(13):1172–83. doi: 10.1056/NEJMoa2107659 PMC8262625 34192426

[41] Bravo L Smolenov I Han HH Li P Hosain R Rockhold F . Efficacy of the adjuvanted subunit protein covid-19 vaccine, scb-2019: A phase 2 and 3 multicentre, double-blind, randomised, placebo-controlled trial. Lancet (2022) 399(10323):461–72. doi: 10.1016/S0140-6736(22)00055-1 PMC8776284 35065705

[42] Chan JF Zhang AJ Yuan S Poon VK Chan CC Lee AC . Simulation of the clinical and pathological manifestations of coronavirus disease 2019 (Covid-19) in a golden Syrian hamster model: Implications for disease pathogenesis and transmissibility. Clin Infect Dis (2020) 71(9):2428–46. doi: 10.1093/cid/ciaa325 PMC7184405 32215622

[43] Jiang RD Liu MQ Chen Y Shan C Zhou YW Shen XR . Pathogenesis of sars-Cov-2 in transgenic mice expressing human angiotensin-converting enzyme 2. Cell (2020) 182(1):50–8.e8. doi: 10.1016/j.cell.2020.05.027 32516571 PMC7241398

[44] Tostanoski LH Wegmann F Martinot AJ Loos C McMahan K Mercado NB . Ad26 vaccine protects against sars-Cov-2 severe clinical disease in hamsters. Nat Med (2020) 26(11):1694–700. doi: 10.1038/s41591-020-1070-6 PMC7671939 32884153

[45] Bricker TL Darling TL Hassan AO Harastani HH Soung A Jiang X . A single intranasal or intramuscular immunization with chimpanzee adenovirus-vectored sars-Cov-2 vaccine protects against pneumonia in hamsters. Cell Rep (2021) 36(3):109400. doi: 10.1016/j.celrep.2021.109400 34245672 PMC8238649

[46] Wu Y Huang X Yuan L Wang S Zhang Y Xiong H . A recombinant spike protein subunit vaccine confers protective immunity against sars-Cov-2 infection and transmission in hamsters. Sci Transl Med (2021) 13(606):eabg1143. doi: 10.1126/scitranslmed.abg1143 34285130 PMC9836081

[47] Lien C-E Lin Y-J Chen C Lian W-C Kuo T-Y Campbell JD . Cpg-adjuvanted stable prefusion sars-Cov-2 spike protein protected hamsters from sars-Cov-2 challenge. Sci Rep (2021) 11(1):8761. doi: 10.1038/s41598-021-88283-8 33888840 PMC8062487

[48] Leventhal SS Clancy C Erasmus J Feldmann H Hawman DW . An intramuscular DNA vaccine for sars-Cov-2 decreases viral lung load but not lung pathology in Syrian hamsters. Microorganisms (2021) 9(5):1040. doi: 10.3390/microorganisms9051040 34065996 PMC8151856

[49] Seephetdee C Buasri N Bhukhai K Srisanga K Manopwisedjaroen S Lertjintanakit S . Mice immunized with the vaccine candidate hexapro spike produce neutralizing antibodies against sars-Cov-2. Vaccines (Basel) (2021) 9(5):498. doi: 10.3390/vaccines9050498 34066016 PMC8151071

[50] Merkuleva IA Shcherbakov DN Borgoyakova MB Isaeva AA Nesmeyanova VS Volkova NV . Are hamsters a suitable model for evaluating the immunogenicity of rbd-based anti-Covid-19 subunit vaccines? Viruses (2022) 14(5):1060. doi: 10.3390/v14051060 35632800 PMC9146860

[51] Ebenig A Muraleedharan S Kazmierski J Todt D Auste A Anzaghe M . Vaccine-associated enhanced respiratory pathology in covid-19 hamsters after T(H)2-biased immunization. Cell Rep (2022) 40(7):111214. doi: 10.1016/j.celrep.2022.111214 35952673 PMC9346010

[52] Winkler ES Gilchuk P Yu J Bailey AL Chen RE Chong Z . Human neutralizing antibodies against sars-Cov-2 require intact fc effector functions for optimal therapeutic protection. Cell (2021) 184(7):1804–20.e16. doi: 10.1016/j.cell.2021.02.026 33691139 PMC7879018

[53] Kingstad-Bakke B Lee W Chandrasekar SS Gasper DJ Salas-Quinchucua C Cleven T . Vaccine-induced systemic and mucosal T cell immunity to sars-Cov-2 viral variants. Proc Natl Acad Sci U.S.A. (2022) 119(20):e2118312119. doi: 10.1073/pnas.2118312119 35561224 PMC9171754

[54] Moss P . The T cell immune response against sars-Cov-2. Nat Immunol (2022) 23(2):186–93. doi: 10.1038/s41590-021-01122-w 35105982

