# Supplementary figures and images for: SARS-CoV-2 spike HexaPro formulated in aluminium hydroxide and administered in an accelerated vaccination schedule partially protects Syrian Hamsters against viral challenge despite low neutralizing antibody responses
Source: Front Immunol. 2023 Jan 23;14:941281. doi: 10.3389/fimmu.2023.941281 (PMC9900178; doi:10.3389/fimmu.2023.941281)

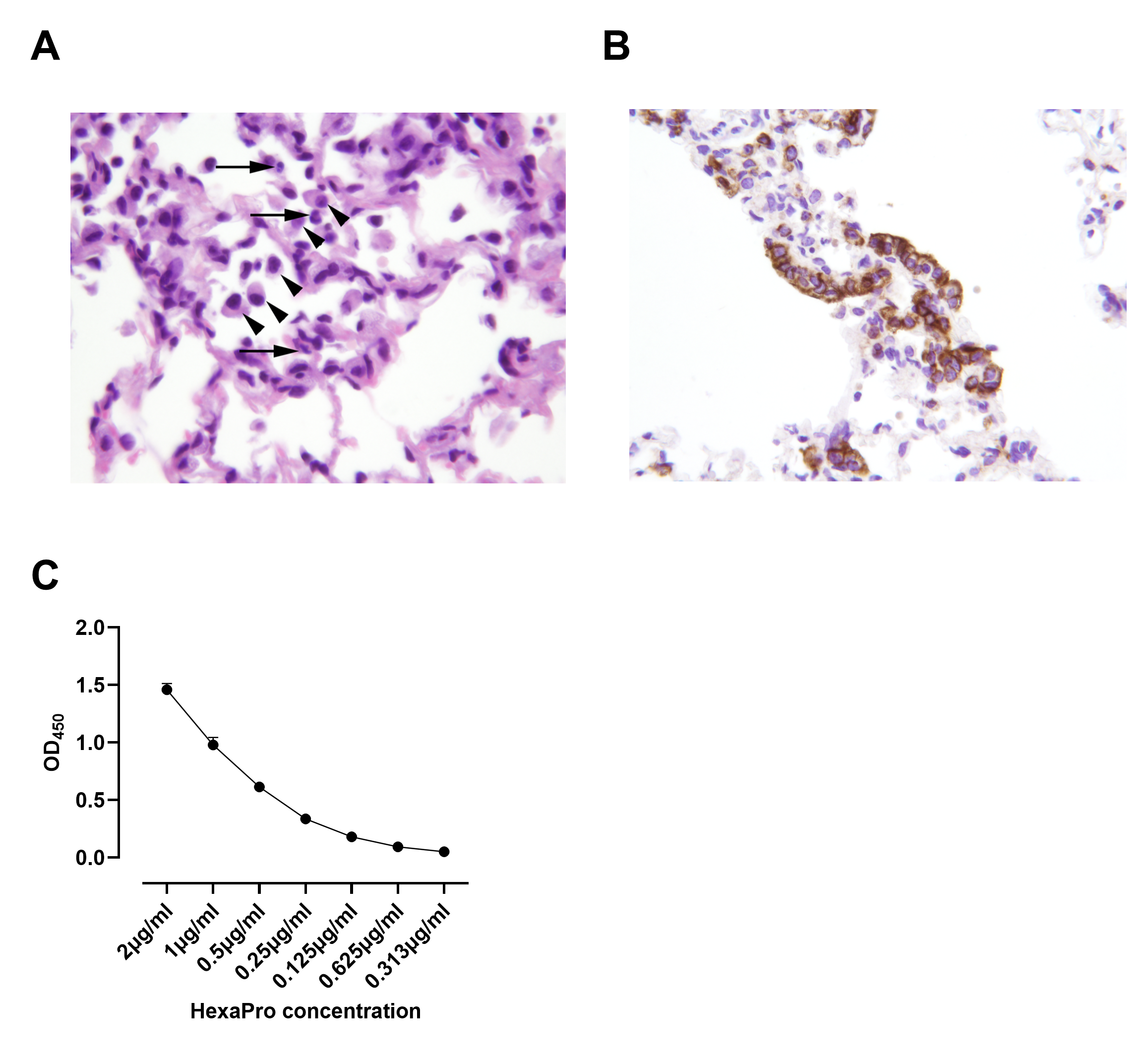

Supplement: Supplementary Figure 1 — (A) Representative stainings of lungs from unvaccinated hamsters at 12 days post infection with SARS-CoV-2. Influx of different inflammatory cells are pointed out. Neutrophils (arrows) and macrophages (arrowheads) are observed. Obj. x 40. (B) Representative stainings of unvaccinated hamsters at 12 days post infection with SARS-CoV-2 for presence of type II pneumocyte hyperplasia, demonstrated by immunostaining of cytokeratin. Obj. x 25. (C) Confirmation of antigen (HexaPro) binding to ACE2 by ELISA. [file Image_1.tif]

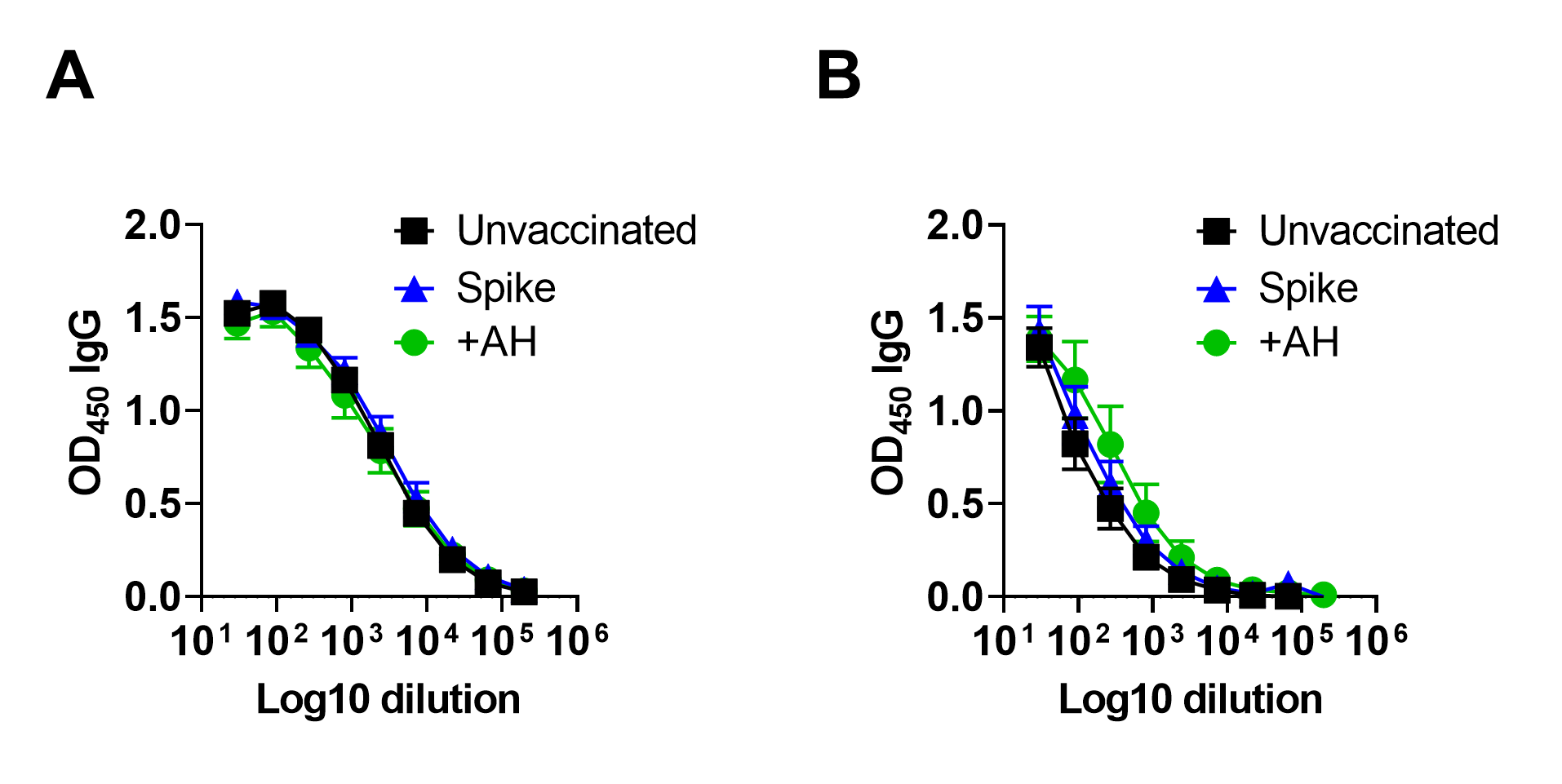

Supplement: Supplementary Figure 2 — Syrian golden hamsters were immunized with two doses of spike trimer protein alone or formulated in AH and challenged intranasally with 1.8x10^5^ TCID50 of SARS-CoV-2. IgG antibody responses were measured in serum from 12 days post-infection against full spike protein from (A) the homologous Wuhan-Hu-1 strain and (B) the omicron (B.1.1.529. variant. [file Image_2.tif]

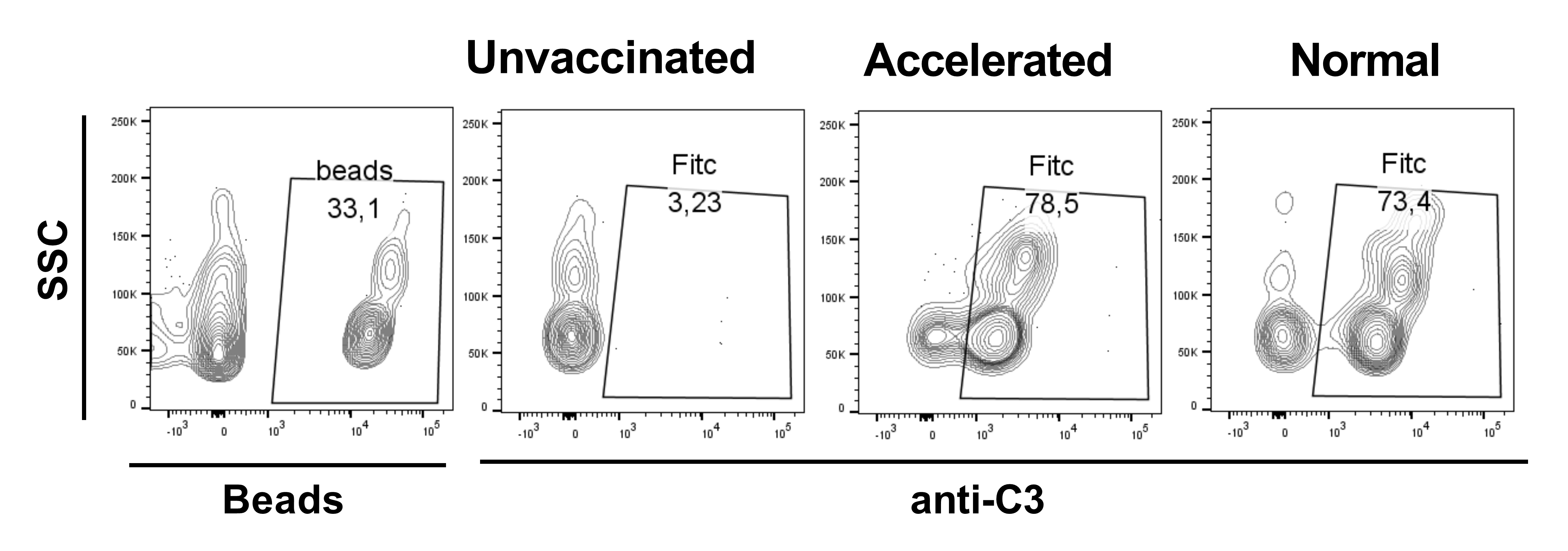

Supplement: Supplementary Figure 3 — Syrian golden hamsters were immunized with two doses of spike trimer protein formulated in aluminium hydroxide given either as an accelerated regimen (10 days apart) and sampled at 11 days after the 2nd immunization or as a normal regimen (21 days apart and sampled 21 days after the 2nd immunization. Antibody dependent complement deposition was measured by coating beads with spike protein. Sera from vaccinated or non-vaccinated hamsters and complement was added and antibody-dependent complement deposition was assayed by measuring C3 deposition by flow cytometry. [file Image_3.tif]
